# Embryonal rhabdomyosarcoma arising from the uterine corpus in a postmenopausal female: a surgical case challenging the genuine diagnosis on a cytology specimen

**DOI:** 10.1186/s13000-016-0451-0

**Published:** 2016-01-12

**Authors:** Sohsuke Yamada, Yoshikazu Harada, Hirotsugu Noguchi, Naoko Satoh, Satoshi Kimura, Toshiyuki Nakayama, Akihide Tanimoto

**Affiliations:** Department of Pathology and Cell Biology, School of Medicine, University of Occupational and Environmental Health, 1-1 Iseigaoka, Yahatanishi-ku, Kitakyushu, 807-8555 Japan; Department of Molecular and Cellular Pathology, Kagoshima University Graduate School of Medical and Dental Sciences, Kagoshima, Japan

**Keywords:** Embryonal rhabdomyosarcoma, Uterine corpus, Cytology

## Abstract

A 55-year-old postmenopausal female presented with genital bleeding and lower abdominal mass. An abdominal MRI revealed a heterogeneously enhanced, 15 × 10 cm mass, completely filling the lumen of the enlarged uterus. The cytologic analysis of the mass showed tumor cells in small clusters and as individual cells showing hyperchromatic round to oval nuclei, and pleomorphic and occasionally unipolar “tadpole”-shaped cytoplasm, in a background of severe necrosis and many degenerated squamous cells. We first interpreted it merely as atypical cells, possibly originated from sarcoma. A total abdominal hysterectomy and salpingo-oophorectomy were performed, and gross examination showed an exophytic polypoid mass with a whitish to white-grayish, necrotic appearance, protruding from the endometrial mucosa. Microscopically, the tumor was composed of a diffuse proliferation of highly atypical spindle-shaped cells, admixed with many characteristic rhabdomyoblasts having abundant densely eosinophilic cytoplasm with sometimes distinct cross-striations, coexisted with cellular primitive small blue round to oval cells foci. However, neither carcinoma nor additional heterologous sarcoma components were completely seen within our thorough investigation. Therefore, we finally made a diagnosis of embryonal rhabdomyosarcoma arising from the uterine corpus. We should be aware that owing to its characteristic features, cytopathologists might be able to determine a genuine diagnosis, based on multiple and adequate cytology samplings.

## Background

Among all adult sarcomas, rhabdomyosarcoma accounts for between merely 2 and 5 % [1, 2]. Also, adult, but not children/adolescents, rhabdomyosarcomas originating from the female genital tracts, including uterus, are very rare and, to date, less than 70 or up to 35 cases of uterine rhabdomyosarcoma have only been reported in the English or Japanese literatures, respectively [1–4]. Uterine rhabdomyosarcoma is histopathologically categorized into three major variants: embryonal; alveolar; and pleomorphic types, and the most common and generally most favorable variant is the embryonal type [1, 2]. In addition, rhabdomyosarcomas are uncommon in patients older than 40 years, even though the most common neoplasm to arise in the uterus is rhabdomyosarcoma, except for leiomyosarcoma and stromal sarcoma [1–3]. Therefore, they often pose a diagnostically big challenge to not only gynecologists but cytopathologists, because it is very hard to obtain the specific cytologic findings, such as cytoplasmic cross-striations or tadpole shape of differentiating rhabdomyoblasts, on an inadequate and small sample, and its entity is difficult to diagnose pre-operatively [5–7]. Uterine rhabdomyosarcomas in adults actually could behave more aggressively and those patients have a relatively worse prognosis due to a higher tendency to develop a recurrence or distant metastases [1–4]. In this context, early accurate diagnosis and radical surgical treatment might be able to improve their survival rates. Herein we reported an extremely rare case of embryonal rhabdomyosarcoma arising from the uterine corpus in a postmenopausal adult female, giving rise to the genuine diagnostic difficulty on an inadequate and small cytology specimen.

## Case presentation

**Fig. 1:**
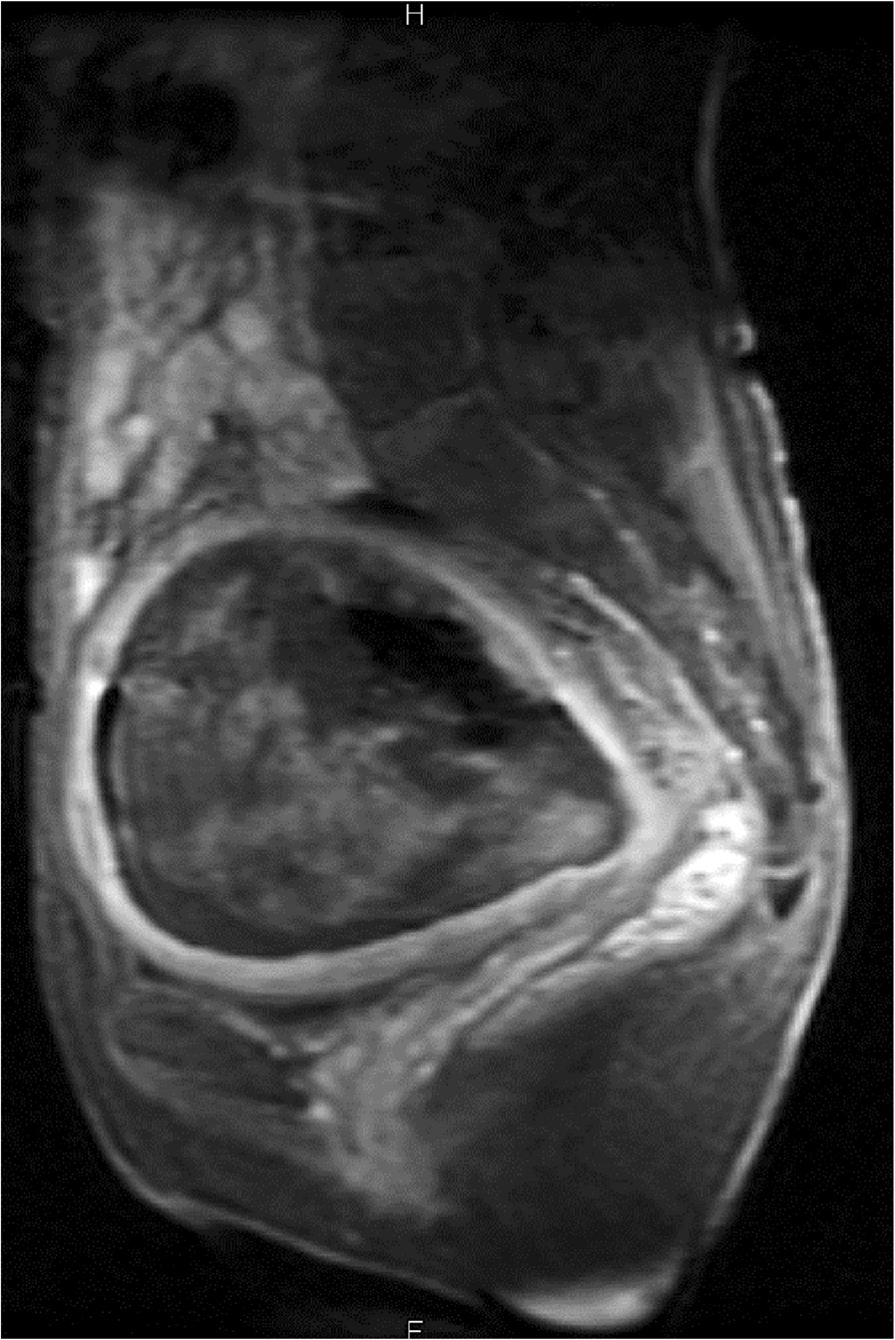
The finding of pelvic MRI at surgery of the uterine rhabdomyosarcoma. The sagittal section of an MRI showed a heterogeneously enhanced huge mass, measuring approximately 15 × 10 cm in diameter, arising possibly from the uterine corpus and, completely filling the lumen of markedly enlarged uterus

The patient presented here, a 55-year-old postmenopausal female (G3P3) with an unremarkable previous medical history, except for appendectomy 30 years ago, had a complaint of dysfunctional uterine bleeding and lower abdominal mass. Laboratory data, including blood cell count, chemistry and tumor markers, were within normal limits. The sagittal section of a pelvic MRI revealed a heterogeneously enhanced huge mass, measuring approximately 15 × 10 cm in diameter, arising possibly from the uterine corpus and, projecting into and completely filling the lumen of markedly enlarged uterus (Fig. 1). Full-body MRI disclosed no definite evidence of metastases in the lymph nodes or other organs. Gynaecological examination showed one part of protruding tumor lesion from the opening of uterus. Clinicians first diagnosed it as a uterine huge leiomyoma.

**Fig. 2:**
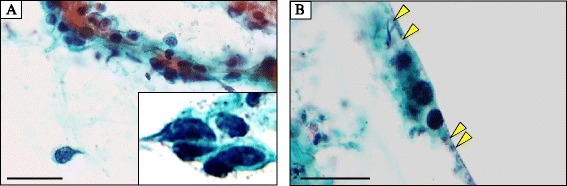
Microscopic examination of the transvaginal brushing cytology specimen from the uterine mass (uterine embryonal rhabdomyosarcoma). **a** The cytologic specimen contained several individual cells or small clusters of atypical tumor cells having hyperchromatic round to oval nuclei, inconspicuous nucleoli, and pleomorphic and occasionally characteristic unipolar “tadpole”-shaped (inset) cytoplasm, in a background of severe necrosis and many degenerated squamous cells. Bar = 25 μm. **b** Retrospectively thorough examination could find out cytoplasmic cross-striations-like structures (arrowheads) in one of atypical spindle-shaped tumor cells. Bar = 25 μm

**Fig. 3:**
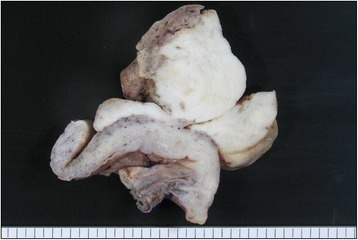
Gross findings of the resected specimen of embryonal rhabdomyosarcoma arising from the uterine corpus. The cut surface showed exophytic polypoid mass lesions with a whitish to white-grayish, necrotic (*left upper*) appearance, measuring more than approximately 11 x 7 cm, protruding from the endometrial mucosa, but not involving the deep myometrium and uterine cervix (*left*)

**Fig. 4:**
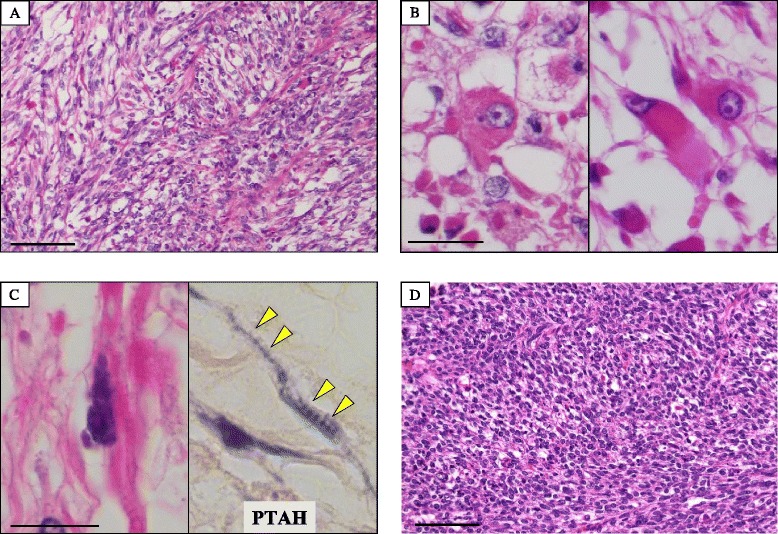
Microscopic examination of the resected embryonal rhabdomyosarcoma arising from the uterine corpus. **a** The viable polypoid lesions were composed predominantly of a diffusely cellular and solid proliferation of highly atypical spindle-shaped cells arranged focally in bundles. Bar = 50 μm. **b** On high-power view, there were many admixed characteristic rhabdomyoblasts having abundant densely eosinophilic, and round (*left*) to tadpole- or strap-shaped (*right*) cytoplasm. Bar = 25 μm. **c** In addition, these atypical cells sometimes contained distinct cross-striations (*left*). Phosphotungstic acid-hematoxylin (PTAH) staining (*right*) was able to clearly reveal these cross-striations (*arrowheads*). Bar = 25 μm. **d** On the other hand, the neoplastic foci partly coexisted with cellular primitive small blue round to oval cells Bar = 50 μm

An endocervical curettage biopsy was performed just before surgery, however, its specimen showed poor study and not diagnostic, since only necrotic tissue was seen. Moreover, the specimen from the transvaginal brushing cytology contained several individual cells or small clusters of atypical tumor cells having hyperchromatic round to oval nuclei, inconspicuous nucleoli, and pleomorphic and occasionally characteristic unipolar “tadpole”-shaped cytoplasm, in a background of severe necrosis and many degenerated squamous cells (Fig. 2a). Retrospectively thorough microscopic examination could find out cytoplasmic cross-striations-like structures in one of atypical spindle-shaped tumor cells (Fig. 2b). We cytopathologists first interpreted it merely as atypical cells, possibly originated from sarcoma, including rhabdomyosarcoma or carcinosarcoma with rhabdoid differentiation, as differential diagnoses. A total abdominal hysterectomy and salpingo-oophorectomy were thus performed, and gross examination displayed an exophytic polypoid mass with a whitish to white-grayish, necrotic appearance, measuring more than approximately 11 × 7 cm, protruding from the endometrial mucosa, but not involving the deep myometrium and uterine cervix (Fig. 3). Resection was diagnosed as complete by our histopathological examination. The covering endocervical epithelium and non-tumor endometrium showed no remarkable change, except for atrophy. Microscopically, its viable polypoid lesions were composed predominantly of a diffusely cellular and solid proliferation of highly atypical spindle-shaped cells arranged focally in bundles with patchy and small acellular foci (Fig. 4a), oppressingly involving the only superficial layer of myometrium. On high-power view, there were many admixed characteristic rhabdomyoblasts having abundant densely eosinophilic, and round to tadpole- or strap-shaped cytoplasm (Fig. 4b) with sometimes distinct cross-striations (Fig. 4c). Phosphotungstic acid-hematoxylin (PTAH) staining was able to clearly reveal these cross-striations (Fig. 4c). Mitotic figures were readily encountered. In addition, these neoplastic foci partly coexisted with cellular primitive small blue round to oval cells (Fig. 4d). By contrast, neither carcinoma nor additional heterologous sarcoma components were completely seen within our thorough investigation. We could not find out any metastatic foci of regional lymph nodes or other organs, either.

**Fig. 5:**
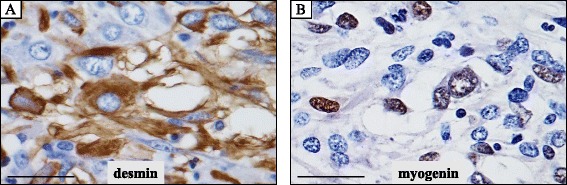
Immunohistochemical examination of the resected embryonal rhabdomyosarcoma arising from the uterine corpus. (**a**, **b**) These highly atypical tumor cells were specifically positive for myogenic markers, such as desmin (**a** cytoplasmic pattern) and myogenin (**b**: nuclear pattern). Bars = 25 μm

All immunohistochemical stainings below were carried out using Dako Envision kit (Dako Cytomation Co., Glostrup, Denmark) according to the manufacturer’s instructions [8, 9]. Immunohistochemically, the atypical tumor cells were specifically positive for desmin (Dako, diluted 1:150) (Fig. 5a), pan-muscle actin (HHF-35; Enzo, New York, USA, diluted 1:20) and myogenin (Dako, diluted 1:30) (Fig. 5b), whereas completely negative for α-smooth muscle actin (α-SMA; Dako, diluted 1:150), caldesmon (Dako, diluted 1:50), CD10 (NOVOCASTRA laboratories Ltd., Newcastle, United Kingdom, diluted 1:20), cytokeratins (AE1/AE3; CHEMICON International, Inc., Temecula, California, USA, diluted 1:200, and Cam5.2; Becton Dickinson Immunocytometry Systems, San Jose, California, USA, diluted 1:1), S-100 protein (Dako, diluted 1:900) and HMB-45 (Enzo, diluted 1:100) (data not shown).

Based on all these features, we finally made a diagnosis of primary embryonal rhabdomyosarcoma arising from the uterine corpus in an adult female. To date, approximately 6 months routine follow-up after the surgery is established, and the patient remains well and no recurrence has been identified.

## Conclusions

Early diagnosis and aggressive clinical treatment for rhabdomyosarcoma of the uterine corpus especially in an adult female could be the hope for better prognosis, due to a higher-grade malignant tumor [1–4], even though, until now, the case number reported as it in the English literatures has been very small and there has been no large series of it, to the best of our knowledge. It is thus critical to establish a genuine preoperative diagnosis by brushing cytology or biopsy, the clinical utility of which in diagnosing uterine tumors has been established very well. However, any findings with regard to cytology/biopsy for the adult rhabdomyosarcoma of the uterine corpus have been very rarely described, since these methods are frequently not successful due to the severe necrosis particularly of its surface [6, 7]. In fact, this is the first single-case English report of embryonal rhabdomyosarcoma arising from the uterine corpus in an adult female, especially focusing on its cytologic findings, within our thorough investigation.

The cytologic features should reflect the histopathological ones resembling the two major rhabdomyosarcoma subtypes, embryonal and alveolar, showing many small clusters of tumor cells or cellular individual tumor cells having hyperchromatic small round to oval nuclei, and pleomorphic and sometimes unipolar “tadpole”-shaped scant cytoplasm, in the background of necrosis and/or hemorrhage, reminiscent of malignant small round cell tumors [5–7]. On the other hand, cytologically cytoplasmic cross-striations are extremely rare [5]. As to the present case, although the specimens were seemingly too small and inadequate to recognize the cytomorphologic variety, the cytology displayed mostly similar to those as described above [5–7]. Furthermore, it is noteworthy that retrospective, careful examination can figure out cytoplasmic cross-striations-like structures in one of atypical tumor cells, as shown in Fig. 2b. Despite that, it is still hard to make an accurate diagnosis of embryonal rhabdomyosarcoma merely based on cytologic specimens, due to sampling errors, lack of experience and/or misinterpretation. In this scenario, multiple and, ultrasound-guided, if possible, brushing/fine needle aspiration (FNA) cytology and biopsy must be performed in cases with a clinical suspicion of uterine sarcoma, including rhabdomyosarcoma. Furthermore, its suspicion should be raised to alert the cytopathologists at the very least, from the side of gynecologists.

An immunohistochemical analysis must also be a nice and helpful guide to reach a genuine cytological diagnosis of embryonal rhabdomyosarcoma arising from the uterine corpus in an adult female. Immunohistochemistry can demonstrate that several myogenic markers, such as desmin, pan-muscle actin (HHF-35) and myogenin, are specifically expressed in those patients’ samples [1, 2, 5], as presented in Fig. 4. Indeed, it is strongly suggested that, when the cytology and biopsy specimens are not enough and not adequate to draw a confident, conclusive diagnosis, immunostaining for those myogenic markers on cytologic smears or cell block preparations can be very useful for the correct interpretation of uterine embryonal rhabdomyosarcoma. Nevertheless, future detailed studies will be further needed to determine whether our indication is significant after collecting and examining a larger number of its cases.

We herein reported a case of an embryonal rhabdomyosarcoma arising from the uterine corpus in an adult post-menopausal female. The present case was tentatively diagnosed as suspicious of sarcoma, not otherwise specified, based on the relatively small and inadequate cytology examination. All cytopathologists should be aware that its cytopathologically characteristic features, including immunohistochemistry, as well as multiple and adequate cytology specimens, might be able to lead to a genuine diagnosis as an embryonal rhabdomyosarcoma from the uterine corpus.

## Consent

Written informed consent was obtained from the patient for the publication of this report and any accompanying images.
